# Gut bacterial tyrosine decarboxylase associates with clinical variables in a longitudinal cohort study of Parkinsons disease

**DOI:** 10.1038/s41531-021-00260-0

**Published:** 2021-12-15

**Authors:** Sebastiaan P. van Kessel, Petri Auvinen, Filip Scheperjans, Sahar El Aidy

**Affiliations:** 1 Host-Microbe Metabolic Interactions, Groningen Biomolecular Sciences and Biotechnology Institute (GBB), University of Groningen, Groningen, the Netherlands; 2 Institute of Biotechnology, DNA Sequencing and Genomics Laboratory, University of Helsinki, Viikinkaari 5D, 00014 Helsinki, Finland; 3 Department of Neurology, Helsinki University Hospital, and Clinicum, University of Helsinki, Haartmaninkatu 4, 00290 Helsinki, Finland

**Keywords:** Parkinson's disease, Microbiology

## Abstract

Gut microbiota influences the clinical response of a wide variety of orally administered drugs. However, the underlying mechanisms through which drug–microbiota interactions occur are still obscure. Previously, we reported that tyrosine decarboxylating (TDC) bacteria may restrict the levels of levodopa reaching circulation in patients with Parkinson’s disease (PD). We observed a significant positive association between disease duration and the abundance of the bacterial *tdc*-gene. The question arises whether increased exposure to anti-PD medication could affect the abundance of bacterial TDC, to ultimately impact drug efficacy. To this end, we investigated the potential association between anti-PD drug exposure and bacterial *tdc*-gene abundance over a period of 2 years in a longitudinal cohort of PD patients and healthy controls. Our data reveal significant associations between *tdc*-gene abundance, several anti-PD medications, including entacapone, rasagiline, pramipexole, and ropinirole but not levodopa, and gastrointestinal symptoms, warranting further research on the effect of anti-PD medication on microbial changes and gastrointestinal function.

## Introduction

In recent years, many studies have focused on the changes in microbiota composition in individuals with Parkinson’s disease (PD) compared to healthy subjects (extensively covered in several systematic reviews^1,2^ among others). While certain differential abundance alterations were reproduced across studies, variation of results remained considerable^1,2^. One of the reasons that may explain the inconsistency among these studies are confounding factors, such as anti-PD medications, disease duration, and gastrointestinal (GI) symptoms. Indeed, studies took these factors into account with variable effort. Catechol-*O*-methyltransferase (COMT) inhibitors, anticholinergics, and potentially levodopa/carbidopa were found to have a significant effect on the changes in the microbiota^3–6^. Apart from medication, GI dysfunction should be considered when analyzing the altered microbiota in PD patients. Indeed, PD patients usually experience more GI dysfunction symptoms compared to healthy controls (HCs)^7,8^ and intestinal transit time can impact microbiota composition^9^.

Moreover, it has been shown that there is an association between anti-PD medication and GI symptoms. For example, anti-PD medications have been associated (corrected for disease duration) with the total GI Symptoms Rating Score, upper GI symptoms, and hypoactive GI functions^8^. Furthermore, COMT inhibitor dosage was significantly higher in patients with an abnormal transit time compared to those with normal transit time^10^. However, the statistical analysis in that study could not distinguish whether levodopa equivalent daily dose (LEDD) or disease duration was the larger contributing factor to slow colon transit^10^. In addition, ex vivo rodent studies and in vivo dog and human studies showed an effect of dopamine agonists and/or dopamine (which can originate from levodopa in PD patients) on gut motility, as recently reviewed^11^ (and citations therein). Gut microbial metabolization of unabsorbed residues of levodopa were also shown to influence ileal motility ex vivo^12^.

Recent studies have shown that tyrosine decarboxylating (TDC) bacteria can decarboxylate levodopa into dopamine in the periphery, thus restricting levodopa availability to the brain^13,14^. Potentially, TDC-harboring bacteria could create a vicious circle, wherein peripheral dopamine production affects gut motility, favoring the colonization of (TDC)-bacteria^13^. Additionally, non-levodopa anti-PD medications (monoaminoxidase inhibitors, COMT inhibitors, and dopamine agonists), which affect the peripheral dopaminergic balance, may lead to an altered GI function, potentially contributing to (TDC)-bacterial overgrowth and ultimately variable bioavailability of levodopa. However, levels of TDC-bacteria have not yet been measured nor previously correlated with GI symptoms in longitudinal PD cohorts.

In this study, we focused on measuring fecal *tdc*-gene abundance and its association with anti-PD medication exposure in a 2-year longitudinal cohort consisting of 67 PD patients and 65 healthy matched subjects, previously used in an investigation of microbiota and PD^4,5^.

## Results

### Clinical variables

Clinical variable comparison between the longitudinal cohort of PD and HCs did not reveal any significant differences in sex, age (at stool collection), or body mass index, with no systemic antibiotics used by either group within the last month (Supplementary Table 1). The duration of motor and non-motor symptom onset in the PD cohort at baseline was ~8 years (Supplementary Table 1). Over time (between baseline and follow-up), the LEDD significantly increased by an average of 116 mg (Table 1). On average, the Unified Parkinson’s Disease Rating Scale (UPDRS) I and II scores significantly increased over time, while UPDRS III (at ON state) significantly decreased (Supplementary Table 2). The latter may be explained by the significant LEDD increase over time. The Hoehn & Yahr (at ON state) score slightly increased over time (Supplementary Table 2).

**Table 1:** Paired tests between follow-up and baseline samples in PD and HCs (significant test results are printed in bold).

	Paried tests (follow-up–baseline)															
	PD							Control							PD on medication	
	Paired mean difference	Baseline mean ± SD (*n*)	Follow-up mean ± SD (*n*)	*T* test	Wilcoxon	McNemar	FDR	Paired mean difference	Baseline mean ± SD (*n*)	Follow-up mean ± SD (*n*)	*T* test	Wilcoxon	McNemar	FDR	Baseline (%)	Follow-up (%)
*tdc*-gene abundance																
* tdc*-gene abundance (outliers removed)	**2.2E−06**	1.5E−6 ± 1.3E−6 (55)	3.7E−6 ± 2.6E−6 (55)		**9.7E−07**		n.a.	8.61E−07	2.2E−6 ± 2.2E−6 (54)	3.1E−6 ± 2.7E−6 (54)		0.167		n.a.		
Gastrointestinal symptoms																
Wexner total score	**0.72**	5.66 ± 4.01 (67)	6.37 ± 4.63 (67)		**0.033**		**0.059**	**−0.54**	2.92 ± 2.61 (65)	2.38 ± 2.58 (65)		**0.010**		**0.030**		
Rome III (constipation and defecation)	−0.01	7.37 ± 5.96 (67)	7.36 ± 5.17 (67)		0.685		0.685	−0.22	2.97 ± 3.60 (65)	2.75 ± 3.60 (65)		0.651		0.977		
Anti-PD medication exposure																
LEDD (mg)	116.40	444.2 ± 315.6 (66)	560.6 ± 294.0 (66)		**1.8E−06**		**3E−05**									
Levodopa																
Levodopa IR (mg)	54.10	99.63 ± 159.46 (67)	153.73 ± 204.92 (67)		**0.028**		0.221								35.8	41.8
Duodopa (mg)	19.33	0.00 ± 0.00 (66)	19.33 ± 157.06 (66)		0.317		0.708								0.0	1.5
Levodopa CR (mg)	−8.96	47.76 ± 157.98 (67)	38.81 ± 115.41 (67)		1.000		1.000								11.9	14.9
Levodopa (with entacapone) (mg)	19.03	88.06 ± 220.17 (67)	107.09 ± 226.33 (67)		0.513		0.872								14.9	22.4
COMT inhibitors																
Entacapone (mg)	49.25	146.27 ± 357.74 (67)	195.52 ± 385.51 (67)		0.319		0.708								14.9	22.4
MAO inhibitors															0.0	0.0
Selegeline (mg)	−0.51	4.10 ± 4.84 (67)	3.59 ± 4.74 (67)		0.348		0.708								43.3	38.8
Rasagiline (mg)	0.06	0.25 ± 0.44 (67)	0.31 ± 0.47 (67)		0.102		0.434								25.4	31.3
Dopamine agonists																
Rotigotine (mg)	0.42	0.00 ± 0.00 (67)	0.42 ± 1.62 (67)		**0.039**		0.221								0.0	7.5
Pramipexole (mg)	−0.06	0.62 ± 0.82 (67)	0.56 ± 0.88 (67)		0.613		0.885								43.3	38.8
Ropinirole (mg)	0.04	3.15 ± 5.18 (67)	3.19 ± 6.02 (67)		0.951		1.000								34.3	29.9
Bromocriptine (mg)	n.a.	0.15 ± 1.22 (67)	0.15 ± 1.22 (67)		1.000		1.000								1.5	1.5
Other medication																
Amantadine (mg)	1.49	6.72 ± 40.73 (67)	8.21 ± 38.53 (67)		1.000		1.000								3.0	4.5
Biperiden (mg)	−0.11	0.21 ± 0.94 (67)	0.10 ± 0.55 (67)		0.345		0.708								6.0	4.5
Exelon (mg)	n.a.	0.00 ± 0.00 (67)	0.00 ± 0.00 (67)		1.000		1.000								0.0	0.0
Use of anacidic (yes/no)	−3%	6/61 (67)	4/63(67)			0.625	0.885	**−11%**	7/58 (65)	0/65 (65)			**0.016**	**0.032**	9.0	6.0
Use of anticholinergic (yes/no)	−4%	6/61 (67)	3/64(67)			0.375	0.708	2%	0/65 (65)	1/65 (65)			1.000	1.000	9.0	4.5

Paired *T* test for normally distributed paired data, paired Wilcoxon test for non-normally distributed paired data, and McNemar for binary distributed paired data. The *p* values were corrected for false discovery rate (FDR) per section.

*n.a.* not applicable.

### Gut bacterial *tdc*-gene abundance, GI symptoms, and medication exposure significantly increased over time in PD patients

It has recently been shown that TDC-bacteria in the GI tract interferes with the availability of levodopa medication in animal models and that longer disease duration and exposure to levodopa may further increase the abundance of TDC-bacteria in the gut^13^. Thus, we sought to investigate the changes in the levels of gut bacterial *tdc*-gene abundance over time in a longitudinal PD cohort, including the differences between PD patients and matching HCs.

**Fig. 1:**
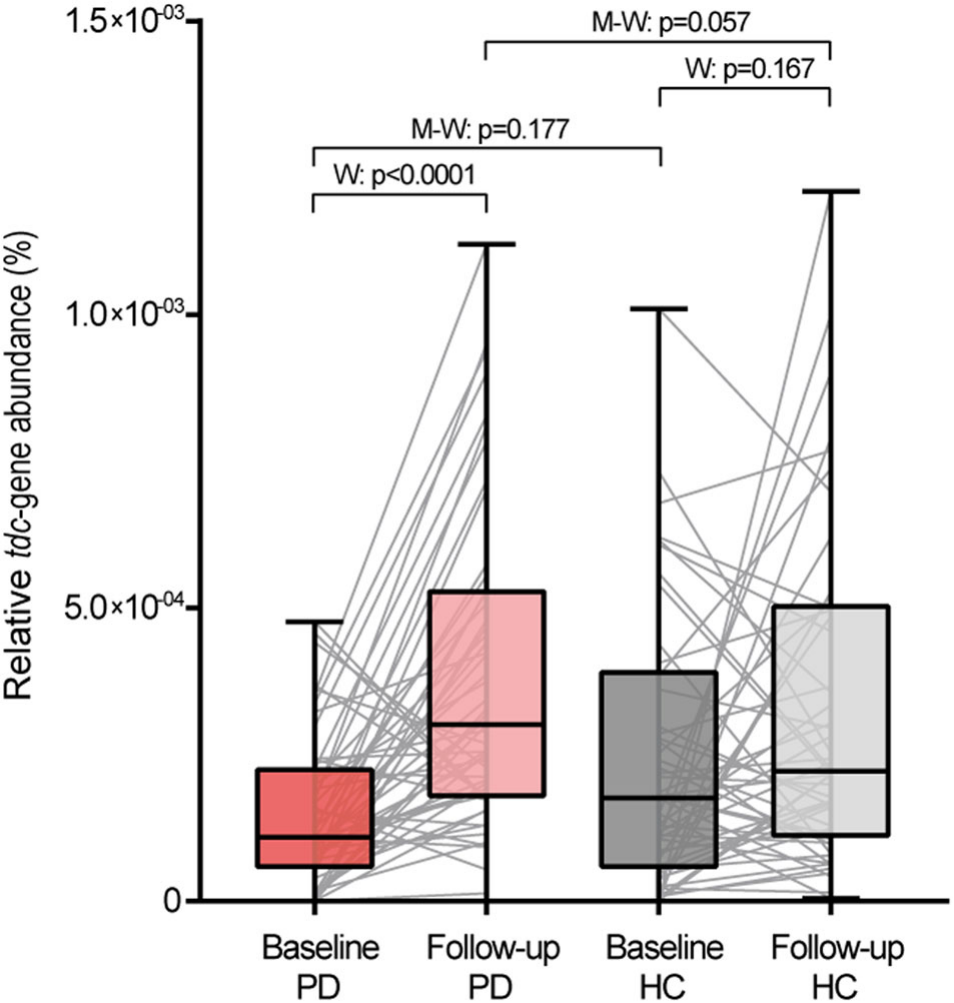
*tdc*-gene abundance in PD and healthy control subjects. The *tdc*-gene abundance is depicted for PD patients (PD, red boxes; dark, baseline; light, follow-up) and healthy control subjects (HCs, gray boxes; dark, baseline; light, follow-up) for both time points. Nonparametric paired Wilcoxon tests (W) were performed to test for significant increase over time between paired samples (gray lines). Significant outliers were removed using the ROUT method (*Q* = 0.1%). Nonparametric unpaired Mann–Whitney tests (M-W) were performed to test for significant differences between PD and HCs at baseline and follow-up. Boxes represent the median with the interquartile range and whiskers the maxima and minima.

High prevalence of the *tdc*-gene was detected at baseline in 97% (*n* = 61/63) and 98% (*n* = 61/62) of the HC and PD samples, respectively. Likewise, at follow-up the *tdc*-gene was detected in 100% (*n* = 64/64) and 98% (*n* = 63/64) of the HC and PD samples, respectively. When comparing PD patients and HCs, PD patients tended to have a higher *tdc*-gene abundance (*p* = 0.057) at follow-up (Fig. 1 and Table 2). Correspondingly, the increase in *tdc*-gene abundance over time was significantly higher in PD patients compared to HC subjects (Wilcoxon test, *p* = 9.7E−07), with a mean increase of 2.6-fold (Table 1 and Fig. 1). The results indicate that, over time, *tdc*-gene abundance increases more rapidly in PD patients compared to HC subjects.

**Table 2:** Independent tests between PD and HCs (significant test results are printed in bold).

	Independent tests (PD–control)													
	Baseline							Follow-up						
	Mean difference	PD mean ± SD (*n*)	Control mean ± SD (*n*)	*T* test	Mann–Whitney	Fisher’s test	FDR	Mean difference	PD mean ± SD (*n*)	Control mean ± SD (*n*)	*T* test	Mann–Whitney	Fisher’s test	FDR
*tdc*-gene abundance														
*tdc*-gene abundance (outliers removed)	−7.4E−07	1.5E−6 ± 1.3E−6 (55)	2.2E−6 ± 2.2E−6 (54)		0.177		n.a.	6.47E−07	3.8E−6 ± 2.6E−6 (57)	3.1E−6 ± 2.7E−6 (55)		0.057		n.a.
Gastrointestinal symptoms														
Wexner total score	**2.73**	5.66 ± 4.01 (67)	2.92 ± 2.61 (65)		**2.4E−05**		**2.4E−05**	**3.99**	6.37 ± 4.63 (67)	2.38 ± 2.58 (65)		**5.9E−09**		**1.2E−08**
Rome III (constipation and defecation)	**4.40**	7.37 ± 5.96 (67)	2.97 ± 3.60 (65)		**1.2E−06**		**2.4E−06**	**4.60**	7.36 ± 5.17 (67)	2.75 ± 3.60 (65)		**1.3E−08**		**1.3E−08**
Medication														
Use of anacidic (yes/no)	−2%	6/61 (67)	7/58(65)			0.777	0.777	6%	4/63 (67)	0/65 (65)			0.119	0.238
Use of anticholinergic (yes/no)	**9%**	6/61 (67)	0/65 (65)			**0.028**	**0.056**	3%	3/64 (67)	1/64 (65)			0.619	0.619

Unpaired *T* test for normally distributed unpaired data, unpaired Mann–Whitney test for non-normally distributed unpaired data, and Fisher’s test for binary distributed unpaired data. The *p* values were corrected for false discovery rate (FDR) per section.

*n.a.* not applicable.

Because GI transit time also impacts microbial composition (including TDC bacteria)^9^, differences in GI symptoms were assessed at baseline and follow-up. At both time points, GI symptoms were significantly more severe in PD patients compared to HC subjects (Table 2). Only the Wexner scores, but not the Rome III scores, increased significantly over time in PD patients, while in HC subjects the Wexner scores decreased significantly over time (Table 1).

Although the LEDD increased significantly over time (Table 1), no significant increase was observed for any individual drug in PD patients after correction for FDR, possibly due to changes in medication type (Table 1). When medication shared between the PD and HC groups was compared at baseline, anticholinergic medication use was significantly higher in PD patients compared to HC subjects (Table 2). In the HC group only, a significant decrease in antacids medication use was observed over time (Table 1).

### Anti-PD medication and GI symptoms associate with the gut bacterial *tdc*-gene abundance over time

Using general linear models (GLMs), the contribution of the difference in anti-PD medication exposure to the difference in *tdc*-gene abundance over time (follow-up–baseline) was assessed (Table 3). The model showed that dose changes of entacapone, rasagiline, pramipexole, and ropinirole significantly contributed to differenential *tdc*-gene abundance over time. Entacapone and the dopamine agonists contributed positively to the difference in *tdc*-gene abundance, while monoamine oxidase inhibitor (MAOi) contributed negatively to the *tdc*-gene abundance over time, respectively.

**Table 3:** General linear model of the difference *tdc*-gene abundance overtime with anti-PD medication and Wexner score as variables (significant variable contributions to the model are printed in bold).

All PD patients (*n* = 55)	Difference *tdc-*gene abundance 2y–0y (no outliers)					
	Not corrected for Wexner score			Corrected for Wexner score		
	*β*	*p* value	VIF	*β*	*p* value	VIF
(Intercept)	**2.1E−06**	**0.000**		**2.5E−06**	**0.000**	
Difference levodopa sum (mg)	1.0E−09	0.671	1.375	−3.2E−10	0.895	1.466
Difference entacapone (mg)	**2.1E−09**	**0.032**	1.163	**2.4E−09**	**0.011**	1.189
Difference selegeline (mg)	−9.4E−08	0.258	1.215	−1.0E−07	0.191	1.218
Difference rasagiline (mg)	**−2.7E−06**	**0.035**	1.366	**−3.0E−06**	**0.013**	1.388
Difference rotigotine (mg)	2.3E−07	0.245	1.161	1.7E−07	0.374	1.184
Difference pramipexole (mg)	**2.0E−06**	**0.001**	1.415	**1.7E−06**	**0.005**	1.515
Difference ropinirole (mg)	**2.2E−07**	**0.028**	1.367	1.6E−07	0.107	1.472
Difference in Wexner total score	Not included			**−3.0E−07**	**0.024**	1.265

*VIF* variance inflation factor.

Because the Wexner scores, but not Rome III, significantly increased over time in the PD group (Table 1), this factor was included in the model to correct for its potential contribution to the *tdc*-gene abundance. Remarkably, Wexner total scores significantly contributed negatively to the *tdc*-gene abundance (Table 3), suggesting that subjects with less constipation have an increased *tdc*-gene abundance. Correction for Wexner scores showed that the difference in exposure to anti-PD medication, stated above, still contributed to the model except for ropinirole (*p* = 0.107). The results indicate that prolonged exposure of these specific anti-PD medications, excluding levodopa, contributed to *tdc*-gene abundance independent of the changes in GI symptoms measured by Wexner scores.

PD patients usually require alterations in their anti-PD dosage regimen during disease progression, compared to patients in a steady state of the disease. Thus, we sought to investigate whether differences in anti-PD dosing between the two groups were a contributing factor to the changes in *tdc-*gene abundance observed above (Table 3). To this end, the PD group was subdivided into slow progressing (*n* = 35) and rapid progressing (*n* = 12) PD patients using the third quartile of the sum of the *z*-transformed changes in UPDRS I-III score (in the ON state) and LEDD between baseline and follow-up as cut-off, as described and performed in Aho et al.^4^. Comparing the mean differences of medications taken by slow and rapid progressing PD patients over time showed that exposure to levodopa and entacapone significantly increased, while pramipexole exposure significantly decreased in the rapid compared to the slow progressing group (Table 4).

**Table 4:** Independent tests between slow and rapid progressing PD patients of exposure of anti-PD medications over time (significant test results are printed in bold).

	Independent tests (rapid–slow progressing)						
	Mean difference	Rapid progressing mean ± SD (*n*)	Slow progressing mean ± SD (*n*)	*T* test	Mann–Whitney	Fishers’s test	FDR
Difference levodopa sum (mg)	**103.5**	208.33 ± 156.43 (12)	104.86 ± 116.32 (36)		**0.022**		**0.051**
Difference entacapone (mg)	**361.1**	366.67 ± 510.50 (12)	5.56 ± 255.17 (36)		**0.009**		**0.051**
Difference selegeline (mg)	1.5	0.83 ± 5.15 (12)	−0.68 ± 4.46 (36)		0.737		0.983
Difference rasagiline (mg)	0.0	0.08 ± 0.29 (12)	0.08 ± 0.28 (36)		0.934		0.983
Difference rotigotine (mg)	0.4	0.83 ± 2.89 (12)	0.39 ± 1.34 (36)		0.983		0.983
Difference pramipexole (mg)	**−0.4**	−0.31 ± 0.65 (12)	0.10 ± 0.58 (36)		**0.019**		**0.051**
Difference ropinirole (mg)	−0.3	−0.58 ± 5.53 (12)	−0.28 ± 3.18 (36)		0.785		0.983

Unpaired *T* test for normally distributed unpaired data, unpaired Mann–Whitney test for non-normally distributed unpaired data, and Fisher’s test for binary distributed unpaired data. The *p* values were corrected for false discovery rate (FDR).

When comparing the slow progressing PD patient group with the rapid progressing PD patient group (Table 5), only entacapone was not associated with *tdc*-gene abundance, and rotigotine now significantly contributed to the model (which was not observed in all PD patients, Table 3). However, the significance was lost when correcting for Wexner score.

**Table 5:** General linear model of the difference of *tdc*-gene abundance over time with anti-PD medication and Wexner score as variables in slow and rapid progressing PD patients (significant variable contributions to the model are printed in bold).

	Slow progressing PD patients (*n* = 36)						Rapid progressing PD patients (*n* = 12)					
	Difference *tdc-*gene abundance 2y–0y (no outliers)						Difference *tdc-*gene abundance 2y–0y (no outliers)					
	Not corrected for Wexner score			Corrected for Wexner score			Not corrected for Wexner score			Corrected for Wexner score		
	*β*	*p* value	VIF	*β*	*p* value	VIF	*β*	*p* value	VIF	*β*	*p* value	VIF
(Intercept)	**2.1E−06**	**0.001**		**2.4E−06**	**0.000**		6.5E−07	0.589		1.3E−06	0.511	
Difference levodopa sum (mg)	3.4E−09	0.436	2.120	2.7E−09	0.506	2.133	7.1E−10	0.868	1.879	−1.2E−09	0.848	4.149
Difference entacapone (mg)	−4.1E−10	0.777	1.164	−2.0E−10	0.884	1.171	**5.2E–09**	**0.000**	1.780	**4.8E−09**	**0.002**	2.665
Difference selegeline (mg)	−1.8E−07	0.053	1.442	**−1.9E−07**	**0.033**	1.445	−2.4E−07	0.163	3.392	−2.6E−07	0.145	3.538
Difference rasagiline (mg)	**−3.5E−06**	**0.006**	1.100	**−3.8E−06**	**0.002**	1.114	−4.0E–06	0.173	2.998	−3.8E−06	0.190	3.045
Difference rotigotine (mg)	**6.1E−07**	**0.038**	1.287	4.5E−07	0.117	1.388	4.8E−07	0.072	2.494	4.3E−07	0.141	3.033
Difference pramipexole (mg)	**2.3E−06**	**0.002**	1.496	**2.4E−06**	**0.001**	1.502	1.8E−06	0.092	2.152	7.6E−07	0.789	14.750
Difference ropinirole (mg)	**5.4E−07**	**0.000**	1.937	**4.5E−07**	**0.003**	2.126	1.3E−07	0.296	1.930	4.3E−08	0.857	7.454
Difference in Wexner total score	Not included			**−2.8E−07**	**0.048**	1.217	Not included			−2.7E−07	0.680	8.843

*VIF* variance inflation factor.

In the rapid progressing PD group, only entacapone contributed significantly to the change in *tdc*-abundance (Table 5). Because the variation inflation factor (VIF, which tests if the variance of a variable increases with another) suggested collinearity between factors in the rapid progressing PD group, DA agonists and MAOi were combined using LEDD calculation^15^. Using the combined variables in the GLM, no collinearity was observed any longer, while entacapone still contributed significantly to the *tdc*-gene abundance (Supplementary Table 3). These results indicate that the difference in drug exposure over time between slow and rapid progressing PD patients (Table 4) reflect their contribution to the *tdc*-gene abundance in the GLMs (Table 5 and Supplementary Table 3). In summary, these observations indicate that the change in exposure to specific anti-PD medications, like entacapone, can be a significant contributing factor to an increase in *tdc-*gene abundance in rapid progressing PD patients. Concomitantly, other anti-PD medications contribute to *tdc*-gene abundance in slow progressing PD patients.

## Discussion

In this study, we have established that gut bacterial *tdc*-gene abundance significantly increases over time in PD patients (Table 1), in line with previous results, where a significant correlation between disease duration and *tdc*-gene abundance was observed^13^. The levels of gut bacterial *tdc*-gene abundance were not significantly different compared to HCs at baseline but close to significant at follow-up (Table 2). Accordingly, the increase in *tdc*-gene abundance was 2.6-fold higher in PD than in HCs, suggesting that the increased gene abundance occurs more rapidly in PD patients. Here, we did not find a significant correlation between levodopa dosage and *tdc*-gene abundance. This discrepancy could be explained by the relatively low proportion of high levodopa dosages in this study. At baseline and follow-up, 19.4% (max 900 mg) and 26.9% (max 875 mg) of the PD patients had a dose higher than 400 mg/day, respectively, while in the previous study^13^ 60% of the PD patients received a dosage higher than 400 mg/day (max 1100 mg).

Using GLMs, we showed that several anti-PD medications other than levodopa contributed significantly to the *tdc*-gene abundance. Importantly, all tested medications (Table 3) affect the (peripheral) dopaminergic system; COMT inhibitors prevent methylation of levodopa, dopamine, and norepinephrine; MAOis prevent dopamine and norepinephrine oxidation; and DA agonists act on dopamine receptors expressed in the gut. Collectively, these medications were recently shown to elicit an effect on GI symptoms^8^. Although GI dysfunction might be caused by the degeneration of enteric neurons, as observed in PD patients with chronic constipation^16^ and reported in an MPTP mouse model for PD^17^, additional dopaminergic medication may impact the GI function even further. Indeed, the Wexner score, which significantly increased over time in PD patients, did not change the associations between anti-PD medication and *tdc*-gene abundance (except for ropinirole exposure) when considered as a confounder. The potential link between changes in GI symptoms, as measured by Wexner score, and anti PD medications are in agreement with the outcome of a comprehensive meta-analysis showing that PD patients on ropinirole did not have a higher risk of constipation compared to placebo, while those on pramipexole had a higher risk of constipation^18^. Unlike the Wexner score, the Rome III (constipation and defecation) score did not change over time in PD patients, which may be explained by the fact that Rome III assesses symptoms retrospectively over a 3-month period and may reduce sensitivity to change. The difference observed between the two questionnaires confirms the need to develop more sophisticated protocols to detect and investigate GI symptoms in PD patients^8^.

Notably, only entacapone exposure in rapid progressing PD patients contributed to fecal *tdc*-abundance. *Enterococcus* (genus consisting of species harboring TDCs) among others were found to be significantly increased only in PD patients treated with entacapone^6^. However, in their study, Weis et al. did not report whether the tested PD patients were on medications such as MAOi or DA agonists, other than levodopa and/or entacapone^6^. Here we show that, in addition to entacapone, other anti-PD medications seem to affect gut bacterial *tdc*-gene abundance (Table 3).

The major limitation of this study is that we determined bacterial *tdc*-gene abundance in fecal samples, which may not be reflective of actual *tdc*-gene levels in the small intestine, the main absorption site of levodopa and other medications. Moreover, the presence of these genes does not necessarily reflect TDC activity.

In summary, the present study implies important associations between anti-PD medication and gut bacterial *tdc*-gene abundance. These associations point toward complex interactions between anti-PD medication, GI symptoms, and gut bacterial *tdc*-gene abundance, which warrants further research.

## Methods

### Cohort

The original age and sex-matched cohort was recruited for a pilot study in 2015 investigating PD and gut microbiota^5^. All subjects were invited to a follow-up on average 2.25 ± 0.20 years later to investigate temporal stability in the PD microbiota^4^. The study was approved by the ethics committee of the Hospital District of Helsinki and Uusimaa. All participants gave written informed consent and the study was registered at clinicaltrials.gov (NCT01536769).

Of the total 165 subjects (77 PD, 88 HCs) recruited at baseline and follow-up^4,5^, 13 subjects (6 PD, 7 HC) were excluded because they did not return for the follow-up study and 20 subjects (4 PD, 16 HCs) were excluded because of various other reasons at baseline or follow-up. In the control group, 1 subject was excluded for a sibling with PD, 3 subjects for having a common cold, 8 subjects for hyposmia (pre-motor PD symptom), 2 subjects for recent surgery, 1 subject had no matching sample, and 1 sample was missing. In the PD group, 1 subject was excluded because of recent surgery, 1 subject had a change in diagnosis, 1 subject because of a sampling handling issue, and 1 subject because of medical history. In total, 33 subjects (10 PD, 23 HCs) were excluded, resulting in 132 subjects (67 PD, 65 HCs) in this study.

The following parameters were assessed as described in the previous studies Scheperjans et al.^5^ and Aho et al.^4^: GI symptoms (Wexner constipation score^19^, Rome III questionnaire^20^), disease severity (UPDRS^21^), and medication exposure.

### DNA extraction

Stool sample collection and DNA isolation were performed in a previous study^4^. Briefly, stool samples were collected by study subjects into collection tubes pre-filled with DNA stabilizer (PSP Spin Stool DNA Plus Kit, STRATEC Molecular) and stored in the refrigerator until transport (for up to 3 days). After receipt of samples, they were transferred to −80 °C. DNA from both baseline and follow-up samples were extracted with the PSP Spin Stool DNA Plus Kit (STRATEC Molecular). Each extraction batch included one blank sample to assess potential contamination. (Of note, to prevent potential technical differences, DNA from baseline samples were extracted at the baseline^5^ and at follow-up^4^, thus the baseline samples were thawed twice.)

### Determination of *tdc*-gene abundance

DNA concentration of samples was directly estimated from 96-well plates by measuring the (pathlength corrected) absorbance at 260 and 320 nm in a multimode reader. The DNA concentration was calculated as follows: 50 × (sample^260–320^ − blank^260–320^). Samples that were negative, very low, or very high in concentration were measured with the nanodrop to confirm. All DNA samples were diluted 20× so that the concentration would fall within the range of 2–25 ng/µl (median, 13.7 ng/µl, interquartile range, 6.9 – 21.8 ng/µl) and 2 µl was used for quantitative PCR (qPCR). qPCR of *tdc* genes was performed using primers Dec5f (5’-CGTTGTTGGTGTTGTTGGCACNACNGARGARG-3’) and Dec3r (5’-CCGCCAGCAGAATATGGAAYRTANCCCAT-3’), targeting a 350 bp region of the *tdc* gene^22^. For primers targeting 16S rRNA gene for all bacteria^23^, Eub338 (5’-ACTCCTACGGGAGGCAGCAG-3’) and Eub518 (5’-ATTACCGCGGCTGCTGG-3’) were used as internal controls for sample bias and total bacterial load. All qPCR experiments were performed in a Bio-Rad CFX96 RT-PCR system (Bio-Rad Laboratories, Veenendaal, The Netherlands) with iQ SYBR Green Supermix (170–8882, Bio-Rad) in 10 μl reactions using the manufacturer’s protocol. qPCR was performed using the following parameters: 3 min at 95 °C; 15 s at 95 °C, 1 min at 58 °C, 40 cycles. A melting curve was determined at the end of each run to verify the specificity of the PCR amplicons. Data analysis was performed using the Bio-Rad CFX Manager 3.1 software. Ct[DEC] values were corrected for sample bias and total bacterial load with the internal control (Ct[16 s]) and linearized using 2^−(Ct^[DEC]^ − Ct^[16s]^) based on the 2^−ΔΔCt method^24^.

### Statistics

All statistical tests were performed in IBM SPSS Statistics version 26. The *p* value adjustments were performed in R version 4.0.0 using p.adjust (*p*-values, “fdr”). The qPCR data were tested for outliers per group and time point using the ROUT method (*Q* = 0.1%) in GraphPad Prism v7 and the identified outliers were removed. Outlier removal was restricted to the qPCR data only. All variables were tested for normality using Kolmogorov–Smirnov and Shapiro–Wilk tests using the Explore function in SPSS. Based on the distribution of data, the differences were tested using the appropriate statistical tests. The group sizes and appropriate statistical tests are indicated in the tables. GLMs were performed using the Generalized Linear Models function in SPSS and the main effects were tested using Wald Chi Square test. Additionally, the VIF was computed to check for potential collinearity between variables.

### Reporting summary

Further information on research design is available in the Nature Research Reporting Summary linked to this article.

## Supplementary information

Reporting Summary

Supplementary Information

## Data Availability

Clinical data are not publicly available due to participant privacy and are available from the corresponding authors on reasonable request.
