# Supplementary material for: Gut bacterial tyrosine decarboxylase associates with clinical variables in a longitudinal cohort study of Parkinsons disease
Source: NPJ Parkinsons Dis. 2021 Dec 15;7:115. doi: 10.1038/s41531-021-00260-0 (PMC8674283; doi:10.1038/s41531-021-00260-0)
Supplement: Supplementary file 2 — Supplementary Information [file 41531_2021_260_MOESM2_ESM.pdf]

Supplementary Table 1. Independent test of the basic variables (Significant test-results are printed in bold)

	Independent-tests (PD - Control)						
	**Mean Difference**	**PD Mean±SD (n)**	**Control Mean±SD (n)**	**T-test**	**Mann-Whitney**	**Fishers's test**	**FDR**
Sex (Female/Male)	0.02%	33/34 (67)	32/33 (65)			1.000	
BMI at baseline (kg/m^2)	0.95	27.2±4.3 (62)	26.2±3.5 (61)	0.181			
BMI at follow up (kg/m^2)	0.71	27.3±4.6 (67)	26.6±3.7 (65)	0.330			
Age at stool collection baseline (years)	0.76	65.4±5.5 (67)	64.6±7.0 (65)	0.486			
Age at stool collection follow up (years)	0.92	67.6±5.5 (66)	66.7±6.9 (64)	0.399			
**Age symptom onset**							
Age at motor symptoms onset (years)		59.5±5.3 (66)					
Age at non-motor symptoms onset (years)		57.7±7.8 (53)					
Duration of motor symptoms (years)		8.2±4.1 (66)					
Duration of non-motor symptoms (years)		10.0±6.9 (52)					

Unpaired T-test for normally distributed unpaired data, unpaired Mann-Whitney-test for non-normally distributed unpaired data, and Fisher’s test for binary distributed unpaired data.

Supplementary Table 2. Paired tests between follow-up and baseline of LEDD and UPDRS scores (significant test-results are printed in bold)

	Paired-tests (Follow-up - Baseline)						
	**Mean Difference**	**Baseline Mean±SD (n)**	**Follow up Mean±SD (n)**	**T-test**	**Wilcoxon**	**McNemar**	**FDR**
UPDRS I	0.63	1.8±1.8 (67)	2.5±1.8 (67)		**0.002**		**0.003**
UPDRS II	1.78	11.6±5.5 (67)	11.6±5.5 (67)		**0.001**		**0.003**
UPDRS III (ON-state)	-3.11	31.9±8.9 (64)	28.8±8.7 (64)	**0.001**			**0.003**
UPDRS III (OFF-state)		na	33.7+10.6 (65)				
UPDRS IV	0.84	2.2±2.2 (67)	3.1±3.2 (67)		**0.008**		**0.010**
H&Y (ON-state)	0.13	2.4±.5 (64)	2.5±0.7 (64)		0.054		0.054
H&Y (OFF-state)		na	2.5±0.8 (64)				

Paired T-test for normally distributed paired data, paired Wilcoxon-test for non-normally distributed paired data, and McNemar's test for binary distributed paired data. The p-values were corrected for False Discovery Rate (FDR) per section.

Supplementary Table 3. General linear model of the difference *tdc*-gene abundance overtime with anti-PD medication LEDD combined and Wexner-score as variables in rapid progressing PD patients (significant results are printed in bold)

	Difference *tdc*-gene abundance 2y-0y (no outliers)					
**Rapid progressing PD patients (n=12)**	Not corrected for Wexner score			Corrected for Wexner score		
	β	p-value	VIF	β	p-value	VIF
(Intercept)	6.8E-07	0.550		1.5E-06	0.273	
Difference levodopa sum (mg)	-4.8E-10	0.913	1.500	-2.3E-09	0.619	1.783
Difference entacapone (mg)	**4.5E-09**	**0.001**	1.414	**4.6E-09**	**0.000**	1.421
Difference MAOi LED (mg)	-1.0E-08	0.534	1.315	-9.6E-09	0.547	1.317
Difference in DA agonist LED (mg)	7.1E-09	0.219	2.383	3.2E-09	0.641	3.601
Difference in Wexner total score	Not included			-3.4E-07	0.323	1.874

VIF; Variance Inflation Factor
